# Fractionation of sulfur (S) in beech (*Fagus sylvatica*) forest soils in relation to distance from the stem base as useful tool for modeling S biogeochemistry

**DOI:** 10.1007/s40808-017-0353-5

**Published:** 2017-08-09

**Authors:** Ondrej Hanousek, Thomas Prohaska, Martin Kulhanek, Jiri Balik, Vaclav Tejnecky, Torsten W. Berger

**Affiliations:** 1 Department of Forest and Soil Sciences, Institute of Forest Ecology, University of Natural Resources and Life Sciences Vienna, Peter-Jordan-Strasse 82, 1190 Vienna, Austria; 2 VIRIS Laboratory, Department of Chemistry, University of Natural Resources and Life Sciences Vienna, Konrad-Lorenz-Strasse 24, 3430 Tulln, Austria; 3 Department of Agroenvironmental Chemistry and Plant Nutrition, Czech University of Life Sciences Prague, Kamycka 129, 165 21 Prague, Czech Republic; 4 Department of Soil Science and Soil Protection, Czech University of Life Sciences Prague, Kamycka 129, 165 21 Prague, Czech Republic

**Keywords:** *Fagus sylvatica*, Sequential extraction, Soil acidification, Stemflow, Sulfur biogeochemistry

## Abstract

The investigation of the fractionation of S compounds in forest soils is a powerful tool for interpreting S dynamics and S biogeochemistry in forest ecosystems. Beech stands on high pH (nutrient-rich) sites on Flysch and on low pH (nutrient-poor) sites on Molasse were selected for testing the influence of stemflow, which represents a high input of water and dissolved elements to the soil, on spatial patterns of sulfur (S) fractions. Soil cores were taken at six distances from a beech stem per site at 55 cm uphill and at 27, 55, 100, 150 and 300 cm downhill from the stem. The cores were divided into the mineral soil horizons 0–3, 3–10, 10–20, 20–30 and 30–50 cm. Soil samples were characterized for pH, C_org_, pedogenic Al and Fe oxides and S fractions. Sequential extraction by NH_4_Cl, NH_4_H_2_PO_4_ and HCl yielded readily available sulfate-S (*RAS*), adsorbed sulfate-S (*AS*) and HCl-soluble sulfate-S (*HCS*). Organic sulfur (*OS*) was estimated as the difference between total sulfur (*ToS*) and inorganic sulfur (*RAS* + *AS* + *HCS*). Organic sulfur was further divided into ester sulfate-S (*ES*, HI-reduction) and carbon bonded sulfur (*CS*). On Flysch, *RAS* represented 3–6%, *AS* 2–12%, *HCS* 0–8% and *OS* 81–95% of *ToS*. On Molasse, *RAS* amounted 1–6%, *AS* 1–60%, *HCS* 0–8% and *OS* 37–95% of *ToS*. Spatial S distribution patterns with respect to the distance from the tree stem base could be clearly observed at all investigated sites. The presented data is a contribution to current reports on negative input–output S budgets of forest watersheds, suggesting that mineralization of *OS* on nutrient rich soils and desorption of historic *AS* on nutrient-poor soils are the dominant S sources, which have to be considered in future modeling of sulfur.

## Introduction

Due to the combustion of fossil fuels, European sulfur (S) emissions and deposition increased steadily since the industrial revolution in the middle of the nineteenth century. As a consequence, sulfur deposition in forested ecosystems of north and central Europe peaked in the early 1980s, reaching, in certain cases, loads of more than 100 kg S ha^−1^ year^−1^ (Prechtel et al. 2001). Legislation to reduce acidifying emissions has taken place at an international level, and, e.g., in Austria, SO_2_ emissions declined by 77% from 1990 (75,000 t year^−1^) to 2013 (17,000 t year^−1^; Umweltbundesamt 2015). Throughfall (plus stemflow) fluxes in beech (*Fagus sylvatica*) forests of eastern Austria declined from 23 to 6 kg S ha^−1^ year^−1^ from 1984 to 2013 (Berger and Muras 2016).

Release of previously stored S delays the recovery of pH of soils and surface waters, because sulfate is the only S output in the leachate. Leaching of sulfate is associated with loss of base cations and soil acidification. Reuss and Johnson (1986) called the time prior the peak of S deposition, when soils adsorbed S, a grace period, since base cations remained available for plant uptake and sulfate adsorption neutralized deposited acids resulting from the replacement of –OH^−^ groups on the soil exchanger surface by SO_4_^2−^. Sulfate adsorption/desorption is a concentration-dependent process, meaning that the amount of adsorbed sulfate increased with increasing sulfate soil solution concentrations in the past but desorption is expected in the future due to declining S deposition and associated decreased sulfate soil solution concentrations. However, a time lag may be expected until this new equilibrium is reached, causing higher sulfate outputs than inputs of forest ecosystems.

In fact, in many regions the chemistry of soils and surface waters has not recovered as expected (Berger et al. 2016; Lawrence et al. 2015). Several calibrated watersheds are currently monitored throughout eastern North America and Europe, reporting net losses of base cations in recent decades as reviewed by Watmough et al. (2005). It is striking that the same authors reported that SO_4_^2−^ export exceeded input at 18 out of 21 studied catchments by 6–76 kg SO_4_^2−^ ha^−1^ year^−1^. This discrepancy may be explained by net desorption of inorganic S and net mineralization of organic S (Likens et al. 2002).

Inorganic S, determined as the sum of readily available (also termed water-extractable; Morche 2008), adsorbed (also phosphate-extractable; Likens et al. 2002) and carbonate occluded SO_4_^2−^ (also HCl-extractable S, Chen et al. 1997) represent in general less than 10% of the total S (Likens et al. 2002; Tisdale et al. 1993; Vannier and Guillet 1994). Organic S compounds represent in general over 90% of the total S in soils (Likens et al. 2002; Tisdale et al. 1993; Vannier and Guillet 1994). The organic S is often divided in two groups: ester sulfates (*ES*; a variety of alkyl-, aryl-, phenol sulfates, etc.) and carbon bonded S (*CS*; amino acids, sulfoxides, etc.; Tabatabai 1996). The mineralization of organic S compounds to SO_4_^2−^ depends on the microbial activity, which is affected by many parameters (soil temperature, humidity, pH, use of soil; Morche 2008). Aside the soil microflora, the type of vegetation growing on the soil determines whether *ES* or *CS* will be mineralized (Morche 2008).

Many biological and chemical processes in the soil are pH-dependent. Mineralization of organic S, as a result of high S plant uptake rates in the past, coupled with increasing soil pHs after the end of the acid rain period, provides additional current sulfate sources. The density of net positive surface charges in the soil (humus, clay minerals, Al and Fe oxides and hydroxides) decreases with increasing soil pH (Likens et al. 2002), expecting a further increased desorption rate of sulfate after acid rain had ceased. Precipitation/dissolution processes of aluminum hydroxy sulfate are expected to release sulfate at higher soil pH as well (Prietzel and Hirsch 1998).

Stemflow of beech represents a high input of water and dissolved elements to the forest soil. Due to the particular canopy structure and bark composition stemflow effects are confined to beech trees only and is negligible for other tree species (Berger et al. 2008). Therefore, deposition of acidifying substances (NO_3_^−^, SO_4_^2−^) has been shown to be significantly higher close to a beech stem than in areas affected by throughfall only (Kazda 1983; Kazda and Glatzel 1984; Koch and Matzner 1993; Sonderegger 1981). Comparison of soil chemistry between the infiltration zone (near the base of the stem) and the “between trees area” in old beech stands by Lindebner (1990) and Rampazzo and Blum (1992) in the “Vienna Woods” proved a significant impact of deposition of atmospheric pollutants: soil acidification, heavy metal accumulation, increased total sulfur contents and loss of the base cations, especially in the infiltration zone. Berger and Muras (2016) estimated that routing 50% of measured stemflow fluxes in 1984 through the soil profile at 27 cm downhill of a beech stem in the “Vienna woods” resulted in a deposition load (throughfall plus stemflow) of 215 kg S ha^−1^ year^−1^ while the input at 300 cm distance from the stem (throughfall only) amounted only 15 kg S ha^−1^ year^−1^. Meanwhile (2013), these loads declined to 30 kg S ha^−1^ year^−1^ (stem base at 27 cm) and 5 kg S ha^−1^ year^−1^ (between trees area at 300 cm distance), respectively. Thus, focusing on the spatial heterogeneity of soil chemistry related to the distance from beech stems enables the study of recovery of differently polluted soil within the same stand. Assuming that increasing soil solution fluxes with decreasing distance from the stem cause a quicker steady state of soil SO_4_^2−^ pools in response to currently decreased inputs, studying soil gradients from the base of the stem to “bulk soil” from the between trees area should enable to better characterize soil acidification recovery dynamics (similar to a false chronosequence), as suggested by Berger and Muras (2016).

Sulfur content and the distribution of S into different fractions were investigated in S-deficient podzolic and chernozemic forest soil profiles (Lowe 1965), in spruce forest soils (Vannier and Guillet 1994) and, to a smaller extent, in hardwood forest soils (Likens et al. 2002). All listed S fractions proved to be of importance for S dynamics and S biogeochemistry (e.g., Chen et al. 1997). As pointed out input–output budgets of sulfate are critical for prediction of soil recovery from acid rain. For that reason the relation between inorganic and organic soil S fractions is crucial which may depend on the applied analytical methods. It is suggested that analyzing the micro-spatial heterogeneity of soil columns downhill of a beech stem enables predictions of soil recovery as a function of historic acid loads and time (Berger and Muras 2016). Hence, we analyzed S fractions at different distances to the stem base of beech at nutrient-rich, high pH (Flysch) and nutrient-poor, low pH (Molasse) sites and hypothesized that high historic S deposition coupled with high water inputs via stemflow result in spatial soil patterns of S fractions. The hypothesis was developed into three specific research questions to address above issues:

1. How are S fractions distributed and related to each other in low pH (nutrient-poor) and high pH (nutrient-rich) forest soils?
2. Are stemflow induced soil changes reflected in spatial patterns of S fractions?
3. Do these patterns match the reported negative S-budgets or recovery from acid rain?

## Materials and methods

### Study sites

Three study sites on two different bedrocks (Flysch and Molasse) each were selected in pure old-growth beech stands. The sites J, E and W on Flysch (Table 1) are located in the Vienna Woods along a distance gradient from the urban area from south–east to north–west (prevailing wind direction during high-pressure episodes). The impact of atmospheric pollutants in the early 1980s on soil characteristics of these sites was documented by Sonderegger (1981) and Kazda (1983). The beech stands B1, B2 and F on Molasse are located in the “Kobernaußerwald”, Upper Austria, and have been extensively studied before (Berger et al. 2006, 2009a, b). The study sites on Molasse are characterized by higher mean annual precipitation (1050 mm) and lower mean annual temperature (7.8 °C) than the sites on Flysch (742 mm, 9.2 °C), as indicated by long term means (1971–2000) of nearby weather stations.

**Table 1:** Forest stand characteristics of pure beech stands at the experimental sites on Flysch and on Molasse

Site	Age years	Stems *n* ha^−1^	Timber volume m^3^ ha^−1^	Basal area m^2^ ha^−1^	Tree height m	Elevation m a.s.l.	Slope °	Aspect (from N to E) °	Coordinates (WGS84)	
									N	E
Flysch										
Windischhütte (W)	150	305	910	60	34	460	17	280	48°17′04″	16°13′42″
Exelberg (E)	120	248	525	40	29	460	13	143	48°14′42″	16°15′14″
Jubiläumswarte (J)	170	167	525	40	28	430	14	151	48°13′13″	16°15′56″
Molasse										
Bradirn 1 (B1)	90	434	434	45	30	610	11	293	48°05′18″	13°14′14″
Bradirn 2 (B2)	120	245	245	25	29	640	15	248	48°05′11″	13°16′42″
Frauschereck (F)	100	385	384	42	28	690	7	315	48°05′35″	13°18′36″

Flysch consists mainly of old tertiary and mesozoic sandstones and clayey marls. Nutrient release from this bedrock is high and, consequently, the prevalent humus forms are mull, indicating quick turnover of the forest litter layer (usually less than 2 cm thickness). All soils of these study sites were classified as stagnic cambisol.

Molasse is composed of tertiary sediments (“Hausruck-Kobernausserwald” gravel), which consist mainly of quartz and other siliceous material (granite, gneiss, hornblende schist, pseudotachylite and colored sandstone). Because of this acidic bedrock with low rates of nutrient release, the dominant soil types are dystric cambisols to podzols. Humus form is acidic moder and the thickness of the forest litter layer varies between 5 and 10 cm, indicating slow turnover and accumulation of nutrients.

### Soil sampling and preparation

Soil samples in the vicinity of one beech stem per site were collected in June 2010 (Flysch) and in October 2011 (Molasse). Soil cores were taken with a core sampler of 70 mm diameter to a depth of 50 cm. Two soil cores were sampled at each of the following six distances from one beech stem per site: at 55 cm uphill (further labeled −55), and at 27, 55, 100, 150 and 300 cm downhill from the stem. The cores were divided into the mineral soil horizons 0–3, 3–10, 10–20, 20–30 and 30–50 cm depth and both replicates were mixed. The samples were sieved (mesh size 2 mm), homogenized and oven dried (105 °C, 24 h). Masses of roots (only one diameter class) in the individual soil horizons did not show clear patterns in relation to the distance to the stem base. Hence, uniform S plant uptake rates are assumed within 3 m distance to the stem. Soil pH was measured in 0.01 mol L^−1^ CaCl_2_, according to ISO 10390:2005. Basic soil parameters, relevant for this study, are listed in Table 2. More details on sampling and soil characterization are given by Muras (2012) and Kaliwoda (2015). Organic C was calculated total C (Wösthoff Carmhomat ADG 8, Germany, ÖNORM L1080) minus C_CaCO3_ (Scheibler method: reaction of carbonates with HCl and volumetric determination of emerging CO_2_ according to ÖNORM L1084). In general, soils on Molasse contained more organic carbon, were characterized by wider C_org_/N_tot_ (mineral soil) and C_org_/S_tot_ ratios (total soil profile), were more acidic, more sandy and less supplied with nutrients than soils on Flysch. Total soil S stocks (S m^−2^ per horizon) were within the same range for all study sites (due to higher bulk densities of loamy to clayey soils on Flysch than of sandy soils on Molasse).

**Table 2:** Soil properties (means of six soil profiles at −55, 27, 55, 100, 150 and 300 cm distance to the base of one beech stem per site) at the study sites on Flysch and Molasse. Ranges of pH and exchangeable base saturation in the mineral soil are minimum and maximum values of 30 soil horizons (6 distances × 5 soil depths)

Site	Horizon	C_org_ kg m^−2^	N_tot_ g m^−2^	S_tot_ g m^−2^	C_org_/N_tot_ ratio	C_org_/S_tot_ ratio	pH (CaCl_2_)	Base sat. %
Flysch								
Windischhütte (W)	Forest floor	0.53	16	0.9	32.9	592		
	0–50 cm	7.46	502	73.3	14.9	102	3.2–3.7	63.0–91.7
	Total soil	7.99	518	74.2	15.4	108		
Exelberg (E)	Forest floor	0.47	18	1.3	26.3	363		
	0–50 cm	8.13	667	76.7	12.2	106	3.6–4.3	88.4–99.4
	Total soil	8.60	685	78.0	12.6	110		
Jubiläumswarte (J)	Forest floor	0.51	17	1.3	29.7	395		
	0–50 cm	10.27	967	101.3	10.6	101	3.5–7.0	95.4–100.0
	Total soil	10.78	984	102.6	11.0	105		
Molasse								
Bradirn 1 (B1)	Forest floor	4.84	240	15.4	20.1	314		
	0–50 cm	10.92	568	94.6	19.2	115	2.8–4.4	3.0–14.8
	Total soil	15.76	808	110.0	19.5	143		
Bradirn 2 (B2)	Forest floor	3.92	183	11.4	21.4	344		
	0–50 cm	10.24	585	73.0	17.5	140	2.9–4.3	4.6–13.6
	Total soil	14.16	768	84.4	18.4	168		
Frauschereck (F)	Forest floor	6.14	278	15.2	22.1	404		
	0–50 cm	6.92	377	68.5	18.3	101	2.9–4.3	3.5–15.5
	Total soil	13.06	655	83.7	19.9	156		

### Determination of sulfur fractions

Inorganic S is determined as the sum of readily available (also termed water-extractable; Morche 2008), adsorbed (also phosphate-extractable; Likens et al. 2002) and carbonate occluded SO_4_^2−^ (also HCl-extractable S, Chen et al. 1997). A sequential extraction is applied to obtain these three inorganic S fractions (Chen et al. 1997; Kulhanek et al. 2011; Lowe 1965; Morche 2008; Shan et al. 1992). Although the extraction processes are not sulfate-specific (it cannot be excluded that hydrophilic organic S compounds are co-extracted), it is not likely that the amount of readily available or adsorbed SO_4_^2−^ is overestimated significantly, as demonstrated by Shan et al. (1992). In the case of carbonate occluded SO_4_^2−^, our preliminary measurements have shown that a sulfate-specific measurement, i.e., ion chromatography (IC), is required to not overestimate this fraction, and, consequently, the total inorganic S amount in accordance to Shan et al. (1992).

Organic S can be calculated as the difference between total S and inorganic S. To divide it into *ES* and *CS*, the soil was reduced by HI as described by Johnson and Nishita (1952), modified by Shan et al. (1992) and applied routinely since then (Chen et al. 1997; Kulhanek et al. 2011; Likens et al. 2002; Morche 2008). All reagents, salts and acids applied were of *pro analysis* grade.

#### Total sulfur (ToS)

The *ToS* was determined according to the ÖNORM L 1096 using LECO SC 444 (LECO Corp., St. Joseph, MI, USA). LECO 302–508 was used as a standard.

#### Soil sulfur extraction

The extraction method as described by Tabatabai (1996) and Kulhanek et al. (2011) was slightly modified (NH_4_^+^ instead of Na^+^ salt solutions were used in this work at the same anion concentrations as used by Kulhanek et al. 2011) and applied for extraction of soil S fractions. Soil was extracted separately for each fraction (not sequentially). Each extraction was repeated three times. Each extract was filtered through a syringe filter (0.45 µm). Double sub-boiled HNO_3_ was used for acidification of extracts to 2% (w/w) HNO_3_ prior to analysis. RTS-1 (CCRMP, Ottawa, ON, CA) certified soil reference material was used for method validation. The reference material was certified for both total S content and adsorbed sulfate. The following S fractions were extracted:

*Readily available sulfate-S* (*RAS*) 1.0 g soil sample was shaken with 6 mL 0.02 mol L^−1^ NH_4_Cl solution for 3 h,

*Phosphate extractable sulfate-S* (*PES*) 1.0 g soil sample was shaken in 6 mL 0.02 mol L^−1^ NH_4_H_2_PO_4_ solution for 3 h,

*HCl-extractable sulfate-S* (*HCE*) 1.0 g soil sample was shaken in 20 mL cold 1 mol L^−1^ HCl for 1 h.

#### HI-reduction of soil sulfur

Samples from one site per bedrock (W on Flysch and B1 on Molasse), representing the lowest and highest soil S_tot_ stocks according to Table 2, were investigated in more details: W and B1 soil samples were reduced by HI as described by Johnson and Nishita (1952), modified by Shan et al. (1992) and applied routinely since then (Chen et al. 1997; Kulhanek et al. 2011; Likens et al. 2002; Morche 2008).

*HI-reducible sulfur* (*HIS)* 0.5 g soil sample was reduced using a HI-reducing agent (Shan et al. 1992). Each sample was processed twice.

#### Sulfur content measurement

Sulfur contents in all extracts were determined (detection limit 0.5 µg L^−1^) by ICP-MS (Element XR, Thermo Scientific, Waltham, MA, USA) operated in medium mass resolution, applying external calibration and internal normalization (In and Sb, both single element ICP standards). However, given amounts of *HCE* (sulfate-S) throughout the paper were determined (detection limit 0.1 mg L^−1^) by means of ion-exchange chromatography (IC; ICS 1600, Dionex, USA) with suppressed conductivity, applying the IonPac AS11-HC (Dionex, USA) guard and analytical columns, and the ASRS 300-4 mm suppressor (Dionex, USA). 30 mmol L^−1^ KOH was used as the effluent (flow rate 1 mL min^−1^).

#### Calculation of soil S species

Adsorbed sulfate-S (*AS*) mass fraction: *AS* = *PES* − *RAS*

HCl-soluble sulfate-S (*HCS*) mass fraction: *HCS* = *HCE* − *PES*

Organic sulfur (*OS*) mass fraction: *OS* = *ToS* − *HCE*

For soil profile imaging, *OS* was further divided into

Ester sulfate-S (*ES*) mass fraction: *ES* = *HIS* − *HCE*

Carbon bonded sulfur (*CS*) mass fraction: *CS* = *ToS* − *HIS*

### Determination of pedogenic Al and Fe oxides

Pedogenic Al and Fe oxides (comprising oxides, oxyhydroxides, and hydrated oxides) were analyzed for the sites W (Flysch) and B1 (Molasse). The dithionite-soluble Fe (Fe_d_) and Al (Al_d_) fractions were determined according to Holmgren (1967): 2.0 g soil sample were mixed with 2.0 g Na_2_S_2_O_4_ and shaken for 16 h in 100 mL 0.3 mol L^−1^ sodium citrate − 1.0 mol L^−1^ NaHCO_3_ (4:1) solution. The contents of the oxalate-soluble Fe (Fe_o_) and Al (Al_o_) were determined according to ÖNORM L 1201: 1.0 g soil sample was shaken in 50 mL 0.2 mol L^−1^ ammonium oxalate − 0.2 mol L^−1^ oxalic acid solution (4:3) for 4 h in the dark. The first 10 mL of the filtered extract were not used for measurement. Contents of Fe and Al in these extracts were analyzed by ICP-OES (ICP-OES, Optima 3000 XL, Perkin Elmer, USA), using external calibration.

### Applied statistics

One-way analysis of variance (ANOVA) and Duncan post hoc analyses were applied using IBM SPSS Statistics 21 software (IBM Corporation, Armonk, NY, USA). Mean values of pH, *RAS, AS, HCS, OS* and *ToS* were evaluated with respect to soil depth and distance from a tree stem. ANOVA and the associated multiple range test was used as the data were normally distributed in most cases. In a few cases (*AS* on Molasse at 100 and 150 cm distance, and pH and *ToS* on Flysch at 30–50 and 0–3 cm, respectively), this precondition for ANOVA was fulfilled after a simple 1/square root transformation of the data. All statements on significance throughout the text are based on applied statistics. Bivariate linear correlation matrices were carried out for all combinations of pH, C_org_, Fe_d_, Fe_o_, Al_d_, Al_o_ and individual S fractions for the sites W (Flysch) and B1 (Molasse) using IBM SPSS Statistics 21 software.

## Results

### Tables and figures

**Fig. 1:**
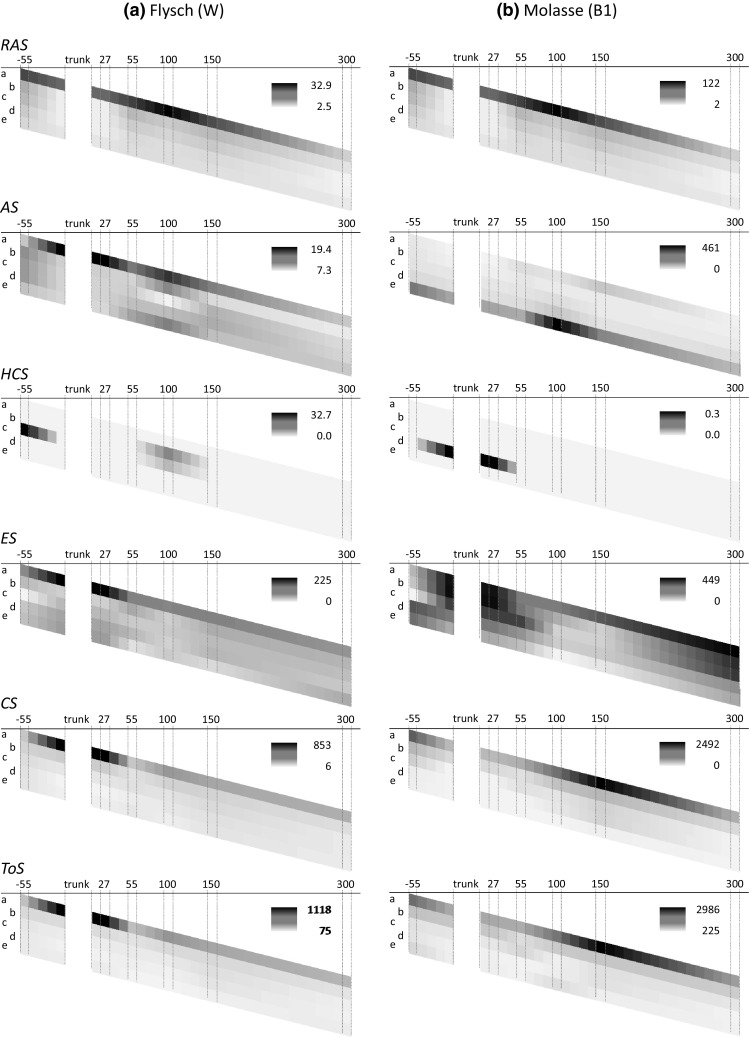
Distribution of readily available sulfate-S (*RAS*), adsorbed sulfate-S (*AS*), HCl-soluble sulfate-S (*HCS*), ester sulfate-S (*ES*), carbon bonded sulfur (*CS*) and total sulfur (*ToS*) on **a** Flysch (study site W) and **b** Molasse (study site B1). Values were measured in the soil horizons 0–3 (*a*), 3–10 (*b*), 10–20 (*c*), 20–30 (*d*) and 30–50 (*e*) cm in −55, 27, 55, 100, 150 and 300 cm distance from a tree trunk. Values between these points were calculated as moving average. Maximum and minimum values are given in the legend of each sub-plot. All data are given in µg S g^−1^

Mean soil pH and sulfur mass fractions in soil profiles at −55 and 27, 55, 100, 150 and 300 cm distance to the base of one beech stem per site are presented for individual horizons on (a) Flysch and (b) Molasse in Table 3. The study sites W (Flysch) and B1 (Molasse), for which *HIS* was measured (enabling the calculation of *ES* and *CS*), were selected for graphical illustration of all possible S fractions in the soil (Fig. 1). The relative S distribution at W (Fig. 1a) and B1 (Fig. 1b) matches well to the mean values of the corresponding S fractions of all three sites on Flysch and Molasse, respectively (Table 3). Bivariate coefficients of linear correlations (*R*, Pearson) among pH, C_org_, Fe_d_, Fe_o_, Al_d_, Al_o_ and individual S fractions in the soil are given for the sites W and B1 in Table 4. Significant (p < 0.01) linear correlations are debated in the “Discussion” section.

**Table 3:**
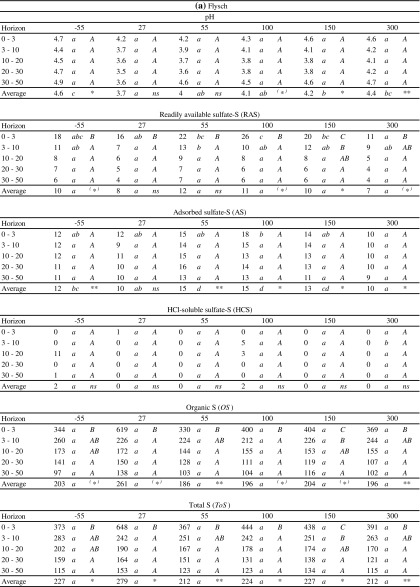

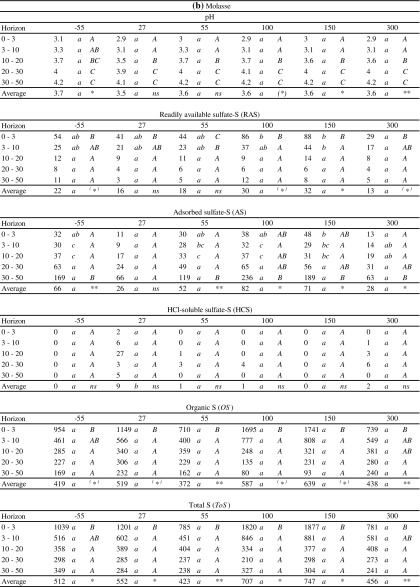
Mean (*n* = 3 sites/trees per bedrock) soil pH (CaCl_2_) and sulfur mass fractions (µg g^−1^) in soil profiles at −55 (uphill) and 27, 55, 100, 150 and 300 (downhill) cm distance to the base of one beech stem per site on (a) Flysch and (b) Molasse

Comparison of means (one-way ANOVA and Duncan multiple range test; 95% confidence interval): small letters for comparison between distances within a given soil horizon and capital letters for comparison between soil horizons within a given profile (*a* or *A* indicates the lowest mean). Average values (*n* = 15), representing mean values of individual soil profiles, were compared between the distances from a tree (small letters) and between Flysch and Molasse (level of significance is shown as: *ns* not significant, p > 0.10; ^(^*^)^p < 0.10; *p < 0.05; **p < 0.01; *n* = 2 bedrocks × 3 sites × 5 horizons = 30)

**Table 4:**
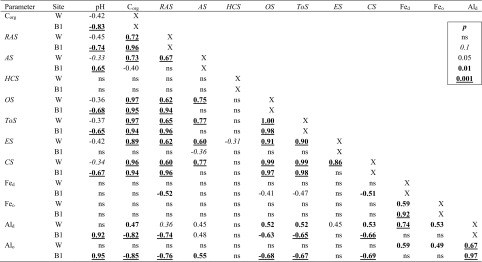
Coefficients of correlation (*R*, Pearson; *n* = 6 profiles × 5 horizons = 30) among pH, C_org_, readily available sulfate-S (*RAS*), adsorbed sulfate-S (*AS*), HCl-soluble sulfate-S (*HCS*), organic sulfur (*OS*), total sulfur (*ToS*), ester sulfate-S (*ES*), carbon bonded sulfur (*CS*), dithionite-soluble Fe (Fe_d_) and Al (Al_d_), and oxalate-soluble Fe (Fe_o_) and Al (Al_o_) at the study sites W (Flysch) and B1 (Molasse)

The level of significance is given as: ns (not significant), italic (*p* < 0.1), normal (*p* < 0.05) bold (*p* < 0.01) and bold plus underlined (*p* < 0.001)

### Soil pH and C_org_

The pH values of mean soil profiles were significantly lower on Molasse than on Flysch, except for the profiles at 27 and 55 cm distance (Table 3).

Organic C contents (C_org_) ranged from 5/21 (30–50 cm depth) to 106/243 (0–3 cm; W/B1, data in mg g^1^) and declined significantly with increasing depth (mean contents of all distances per depth were compared by posthoc Duncan multiple range test; p < 0.05). The mean (*N* = 6 horizons × 6 soil depth = 30) C_org_ content was significantly (p < 0.001) lower at W (20 mg g^−1^) than at B1 (76 mg g^−1^). Though there was a clear trend of enriched C_org_ contents in the soil profile at 27 cm on Flysch (W), no significant impact of distance on C_org_ patterns was recorded (data not shown).

### Sulfur fractions

On average (mean geometric horizon data of three study sites), the *RAS* fraction represented 3–6%, *AS* 2–12%, *HCS* 0–8% and *OS* 81–95% of *ToS* on Flysch. On Molasse, *RAS* amounted 1–6%, *AS* 1–60%, *HCS* 0–8% and *OS* 37–95% of *ToS*.

The contents of all S fractions were significantly higher on Molasse than on Flysch (see statistics for each average soil profile in Table 3), with the exception of not significant differences for *RAS* (27, 55), *AS* (27) and *HCS* (all distances). On both bedrocks, distance from the stem base had no significant effect on *ToS* and *OS* fractions. However, these fractions declined with increasing soil depth (Table 3).

Significantly higher *RAS* values were found in the topsoil on both bedrocks. The *RAS* maxima were reached at distances around 100 cm from the stem base. The *AS* fraction increased with depth on Molasse, while *AS* tended to decline (not significantly) with depth on Flysch. Average *AS* of the soil profile next to the stem base (27 cm) was significantly reduced on Flysch. A similar behavior was observed on Molasse (not significant differences, see Table 3). The *HCS* fraction was significantly enriched next to the stem base (27 cm) on Molasse. Since 1 mol L^−1^ HCl represents the strongest extractant whithin this study, it is suggested that the generally very low *HCS* contents, measured by IC (0–8% of *ToS;* Table 3) represented the remaining sulfate adsorbed on soil particles that was not exchanged by phosphate.

### Pedogenic Al and Fe oxides

Dithionite-soluble Fe (Fe_d_) amounted 3.8–6.6 and 5.5–14.4 mg g^−1^ at W and B1, respectively. The mean Fe_d_ content was significantly (p < 0.001) lower at W (4.6 mg g^−1^) than at B1 (10.8 mg g^−1^). Oxalate-soluble Fe (Fe_o_) amounted 2.3–3.3 and 2.5–14.0 mg g^−1^ at W and B1, respectively. The mean Fe_o_ content was significantly (p < 0.001) lower at W (2.7 mg g^−1^) than at B1 (9.9 mg g^− 1^). The higher Fe_o_/Fe_d_ ratio at B1 (0.9) than at W (0.6) indicated higher crystallinity and lower amounts of more active forms of Fe (Rampazzo et al. 1999). Duncan multiple range tests showed that the content of the mean soil profile at 27 cm was higher than at 55 cm for Fe_d_ and higher than at all other distances for Fe_o_ on Flysch (W), but there was no effect by distance on Molasse (B1; data not shown).

Dithionite-soluble Al (Al_d_) amounted 1.1–1.9 and 2.2–9.7 mg g^−1^ at W and B1, respectively. The mean Al_d_ content was significantly (p < 0.001) lower at W (1.4 mg g^−1^) than at B1 (5.7 mg g^−1^). Oxalate-soluble Al (Al_o_) amounted 0.8–1.2 and 1.6–7.6 mg g^−1^ at W and B1, respectively. The mean Al_o_ content was significantly (p < 0.001) lower at W (1.0 mg g^−1^) than at B1 (4.8 mg g^−1^; data not shown). It must be pointed out that Al_o_ and Al_d_ contents did not differ between mean soil depths at W but increased significantly with increasing depth at B1 (Al_d_: 0–3, 3–10 < 10–20 < 20–30, 30–50; Al_o_: 0–3, 3–10 ≤ 20–30, 30–50; soil horizon ranges in cm).

## Discussion

### How are S fractions distributed and related to each other in low pH (nutrient-poor) and high pH (nutrient-rich) forest soils?

Inorganic SO_4_^2−^ accounts generally for less than 10% of the total S (Tisdale et al. 1993). In the presented study, however, these theoretical limits were exceeded on both investigated bedrocks for mean horizon values (Table 3): the sum of *RAS, AS* and *HCS* reached up to 19 and 63% in some points on Flysch and Molasse, respectively. The *AS* fraction contributed mainly to these high values. According to a simplified S flow diagram of an upland forest ecosystem by Reuss and Johnson (1986), SO_4_^2−^ is the only S output in the leachate. In turn, the SO_4_^2−^ soil solution pool is connected to two S pools: the adsorbed SO_4_^2−^ pool and the organic S pool. In accordance with Reuss and Johnson (1986), we suggest that the observed high inorganic SO_4_^2−^ pools exert a strong control on leaching of SO_4_^2−^ and associated loss of base cations and soil acidification.

Sulfur contained in soil organic matter (*OS*) represented large pools of up to 95% of total S (*ToS*) on both bedrocks which is in accordance with data reported by Vannier et al. (1993) and Erkenberg et al. (1996). The *ES* fraction amounted 38 and 54% of *OS* at W and B1 (mean of average soil profiles), respectively. This is in contradiction to Havlin et al. (2005), suggesting that *ES* represents the majority of the organic S. However, data of *ES* (33 and 39% of *ToS* on W and B1, respectively) are higher than reported contributions of 16% by David et al. (1982) in forest soils, but close to the 38% contribution as found at the Hubbard Brook Experimental Forest (Likens et al. 2002). The total S pool of a haplic podzol in the control watershed Schluchsee consisted of >90% *OS*, 64% of *ToS* was *CS*, and 28% *ES*. However, in a dystric cambisol in the control watershed Villingen, inorganic S bound in the mineral soil accounted for 67% of the *ToS* pool (Prietzel et al. 2001).

Bivariate Pearson correlation coefficients (*R*) are given in Table 4. Caution must be exercised when interpreting correlations because they do not give information about “controlling factors or processes”. Conclusions may be drawn from cases where the coefficients for W and B1 were very contrary. e.g., high positive correlation between *OS* and *AS* at W (0.75; p < 0.001) may be explained by high mineralization rates on Flysch, while the lack of this relation (even negative sign) at B1 may indicate that immobilization (microbial uptake) is an important process on Molasse. Ester sulfate-S, that is considered to be easily mineralized after hydrolysis (Morche 2008), was positively correlated with *AS* at W (indicating mineralization on Flysch) but vice versa at B1 (indicating immobilization on Molasse).

Bivariate relations among S fractions, which were similar for both sites, can be summarized as follows (Table 4): Organic sulfur increased with decreasing pH since acidic conditions promote the accumulation of organic matter and vice versa (i.e., organic matter accumulation produces organic acids). Organic sulfur was positively correlated with all other S fractions at both sites, except with *AS* at B1 (see assumption on preferential S immobilization on Molasse above), *HCS* (probably due to the fact that most data were zero) and with *ES* at B1 (which was distributed more patchy than at W; see Fig. 1). Extremely high coefficients were recorded for both sites between *OS* and *ToS* (W: 1.00; B1: 0.98), which is not surprising as *OS* represents in total the major S fraction in the investigated soils.

Organic C was positively related to *RAS, OS* and *ToS* (p < 0.001) stating the general link between C and S in terrestrial ecosystems (Table 4). The facts that C_org_ was (a) positively related to *AS* at W but negatively at B1, and (b) positively to *ES* at W but not significant at B1 is striking. Possible interpretations are suggested below.

Plant debris (C_org_) provided *CS* at both substrates (*R*, C_org_/*CS*: 0.94–0.96). Carbon bonded S seemed to be rather stable at B1 (*R, CS*/*ES*: ns; suggesting immobilization) but contributed to labile *ES* at W1 (*R, CS*/*OS*: 0.86; suggesting mineralization). According to Ghani et al. (1991), carbon-bonded S is converted, probably oxidatively, to *ES* and is then mineralized to SO_4_^2−^. Labile *ES* probably contributed to *RAS* at W (0.62; note: R, *ES*/*RAS* at B1: ns) which, finally, showed up as adsorbed sulfate-S at W (*R, RAS*/*AS*: 0.67 vs ns at B1). This underlines the suggestion that *AS* originates mainly from decomposition (mineralization) of organic material at Flysch (see high accumulation of *AS* in the top soil of Fig. 1) while *AS* is accumulated in the deep soil at Molasse (Bs horizons with high contents of Al and Fe oxides) as visible in Fig. 1. The fact that a considerable proportion of atmospherically deposited SO_4_^2−^ may be cycled through the organic S pool before being released to soil solution, as suggested for the Flysch sites, was indicated by ^34^S/^32^S ratios (Alewell et al. 1999; Zhang et al. 1998). Selected water samples of rainwater (precipitation and throughfall) and soil solution at the study site W were measured for δ^34^S_VCDT_ by Hanousek et al. (2016) and ranged between 4 and 6‰, the ratio in soil solution being slightly lower. The lower ratio indicated that a considerable portion of the atmospherically deposited sulfate was cycled through the organic S pool before being released to the soil solution and supports our conclusions for the site W above, deduced from relations between the different S-fractions.

Interpreting bi-variate correlation coefficients of Table 4 is difficult due to many inter-correlated variables. e.g., we would expect a positive relation between Al_d_ and C_org_ at B1 (which was negative: *R* = −0.82; p < 0.001) as was recorded at W (*R*: 0.47; p < 0.01), since C_org_ was coupled with negative soil pH (*R*: −0.42 and −0.83 at W and B1, respectively) promoting the formation of Al oxides via silicate weathering (Rampazzo et al. 1999). Because Fe and Al oxides increased significantly with increasing depth at B1 (Bs horizons of podzols on Molasse) but C_org_ contents decreased (similar to depth profiles of *OS*, Table 3) this relation Al_d_ (Al_o_) to C_org_ was negative. As a consequence, the relations of Al_d_ (Al_o_) to *RS* (readily available S is mainly provided from the humus layer), *CS, OS* and *ToS* were all negative as well. However, Al oxides played a major role for adsorption of *AS*, as indicated by positive correlations at B1 (R, Al_d_/*AS* 0.48, p < 0.05; Al_o_/*AS* 0.55, p < 0.01). At W (Flysch), there was a declining trend of Al_d_ with increasing depth, causing significant positive relations to C_org_ and all other S-fractions (except *HCS*). This is why, *AS* was correlated negatively with soil pH (*R* = −0.33) and positively with C_org_ (*R* = 0.73) at W (due to increasing positive charges of humus particles and Al oxides for adsorbing sulfate) but positively with soil pH (*R* = 0.65) and negatively with C_org_ (R = −0.40) at B1 (due to increasing Al oxides with increasing depth at increasing soil pH but decreasing C_org_ contents).

Dithionite-soluble Fe_d_ at B1 showed similar coefficients as Al_d_, though relations with C_org_ and *AS* were not significant (Table 4). At high pH-soils on Flysch (W), the formation of Fe oxides was lower and its role to adsorb sulfate was negligible.

### Are stemflow induced soil changes reflected in spatial patterns of S fractions?

The average pH values of the six total soil profiles per site indicated clearly soil acidification at near downhill distances on Flysch but not on Molasse (Table 3). As soil pHs were lower on Molasse, stemflow induced acidification was not significant as a consequence of the logarithmic pH-scale, of lower base contents (compare Table 2), which do not allow redistribution of large amounts of base cations, and of buffering by the aluminum system at soil pH below 4.2.

Wide soil pH ranges (3.2–7.0) on Flysch (pH: W < E < J; Table 3) are the reason that pH did neither differ with depth within individual soil profiles nor with distance from the stem within one soil depth. On Molasse, at relatively narrow soil pH ranges (2.8–4.4), top soil acidification was recorded for the average soil profiles at all six distances as related to podzolization (Table 3).

Changes within an individual horizon over distance and vice versa (Table 3) were compared to disentangle the soil depth effect on S fractions from stemflow induced spatial patterns. There was no significant effect of stemflow (distance) on distribution of *ToS* and *OS* within the mineral soil, however, total S and *OS* (both for *ES* and *CS* at W) tended to be higher at 27 cm distance in the top soil of Flysch (Table 3a; Fig. 1a). Total S and *OS* showed a general decline with increasing depth. This decline was steeper on Molasse (Table 3b) due to surface accumulation of organic matter on podzolic soils, characterized by slower decomposition and mineralization rates than on Flysch.

Mean contents of total S of 97 beech sites in the Vienna Woods decreased markedly in the stemflow area (0–5 cm soil depth; 20 cm downhill from the base of the stem) but slightly increased in the between trees area (0–5 cm soil depth; at least 3 m away from beech stems) from 1984 to 2012 (Berger et al. 2016), corresponding to a S loss (0–5 cm horizon) of 18.2 g m^−2^ and a S gain of 8.2 g m^−2^, respectively (unpublished data by the same authors). Therefore, high amounts of atmospherically deposited SO_4_^2−^, partly cycled through the organic S pool, must have been mineralized on Flysch since the 1980s. Berger et al. (2009a, b) concluded for comparable beech sites on Flysch that net mineralization of S in the top soil is the major reason for negative SO_4_^2−^ input - output budgets. Preferential mineralization over immobilization on Flysch was suggested from the correlation analysis (Table 4). Narrow mineral soil C_tot_/S_tot_ ratios on Flysch (Table 2) support this conclusion.

Net immobilization of S on Molasse may be another reason why no acidifying effects of stemflow were visible (Table 3b). In fact, SO_4_^2−^ stemflow fluxes caused an accumulation of *ES* along the tree stem (Fig. 1b). Wide mineral soil C_tot_/S_tot_ ratios on Molasse (Table 2) support the theory of high microbial S immobilization rates. High amounts of precipitated Al^3+^ and SO_4_^2−^ as Al hydroxy sulfates in the stemflow area (27 cm) were likely reflected via HCl extractions (as indicated as *HCS* in Table 3b and Fig. 1b), since, according to Prietzel and Hirsch (1998, 2000), phosphate extraction (*AS*) may underestimate inorganic S in acidic soils. A high rate of precipitation/dissolution of Al hydroxy sulfates in the stemflow area would be another reason for buffering acidic input via stemflow.

Desorption of SO_4_^2−^ in response to input of high amounts of water with low SO_4_^2−^ concentrations at the stem after the end of the 1980s is put forward to explain significantly reduced *AS* at the stem base (27 cm) on Flysch (Table 3a). A similar (not significant) trend was visible on Molasse (Table 3b). This trend can be explained by “natural water extractions” (i.e., desorption) over historic time-periods.

### Do these patterns match the reported negative S-budgets or recovery from acid rain?

Contents of *AS* were much lower on the high pH soils on Flysch than on the low pH soils on Molasse, since the density of net positive surface charges decreases with increasing soil pH. Hence, the conclusion by Berger et al. (2009a, b) that top soil mineralization of organic S is the major source of net SO_4_^2−^ output on Flysch agrees with our data. The mean 2-year (2005–2007) net SO_4_-S balance on comparable beech stands (between trees area) on Flysch was estimated −2.0 kg S ha^−1^ year^−1^ (Berger et al. 2009a).

The fact that *AS* increased with depth on Molasse can be explained by the vertical wash-out direction (desorption starts at the top soil) and increasing amounts of Fe- and Al oxides or hydroxides in deeper soil layers (adsorption in the deeper soil; Table 3), as stated in the results section. Reuss and Johnson (1986) pointed out that a long time may be required for passage of the SO_4_^2−^ front to any particular soil depth. It is striking that the soil profile next to the stem (27 cm) is the only one, which did not show a significant vertical increase of adsorbed SO_4_^2−^ (*AS*). It can be suggested that this passage from top to deeper soil horizons has ceased due to accelerated recovery after the reduction of S deposition. Solute SO_4_^2−^ flux profiles (between trees area) for the same beech sites on Molasse by Berger et al. (2009a) showed a steady increase with depth. Hence, desorption of historically deposited S below 10 cm can be assumed as the major source of net S loss for these acidic soils. The mean 2-year (2005–2007) net SO_4_-S balance on the same beech stands on Molasse (between trees area) of this study was estimated −1.4 kg S ha^−1^ year^−1^ (Berger et al. 2009a).

**Fig. 2:**
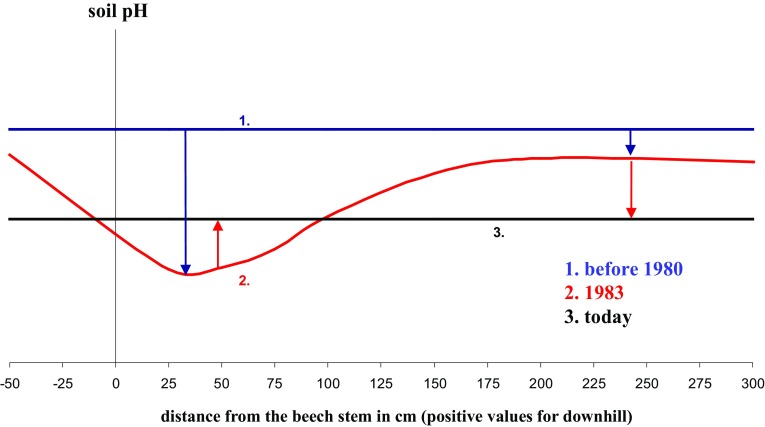
Generalizing spatial soil recovery of the top soil, expressed as pH change (redrawn from Berger and Muras 2016)

In accordance to Reuss and Johnson (1986), it can be concluded that increasing soil solution fluxes with decreasing distance from the stem cause a quicker steady state of soil SO_4_^2−^ pools in response to currently decreased inputs. The presented data on *AS* support this hypothesis. Comparison of old and recent top soil pH values indicates a higher increase in the infiltration zone of beech stemflow than in the “between trees area” (Berger et al. 2016). The data (Table 3) did not show any horizontal pH depression close to the stem, as reported for Flysch sites in the Vienna Woods in the 1980s (Berger and Muras 2016; Kazda 1983; Sonderegger 1981). In contrast to the reported negative S budgets for whole forested watersheds, the stem area of beech seems to have recovered from acid rain - if recovery is defined as the state where input–output S budgets turn from negative to zero. It is concluded that reduced atmospheric sulfate inputs affected soil conditions. Our results match nicely with predictions by Berger and Muras (2016) that the top soil will recover from acid deposition, as already recorded in the infiltration zone of stemflow near the base of the stem. However, in the between trees areas and especially in deeper soil horizons recovery may be highly delayed (Fig. 2).

## Conclusions

Fractionation of S compounds in forest soils is a powerful tool for interpreting S dynamics and S biogeochemistry in forest ecosystems. Stemflow resulted in spatial soil patterns of S fractions (overall hypothesis). On Flysch, *RAS* represented 3–6%, *AS* 2–12%, *HCS* 0–8% and *OS* 81–95% of *ToS*. On Molasse, *RAS* amounted 1–6%, *AS* 1–60%, *HCS* 0–8% and *OS* 37–95% of *ToS*. It was possible to discuss relations between S fractions in regard to important soil processes, e.g., mineralization/immobilization and adsorption/desorption (question 1). Stemflow clearly caused spatial soil patterns of the inorganic S fractions (question 2). Desorption of SO_4_^2−^ in response to input of high amounts of water with low SO_4_^2−^ concentrations at the stem after the end of the 1980s is put forward to explain reduced *AS* at the stem base. Our data contribute to current reports on negative input–output S budgets of forest watersheds, suggesting that mineralization of *OS* on nutrient rich soils and desorption of historic *AS* on nutrient-poor soils are the dominant S sources (question 3), which have to be considered in future modeling of sulfur. Finally, we conclude that the impact of heavy S deposition loads via stemflow and recovery from these inputs can be fingerprinted from the S fraction distribution in the soil. We suggest that combining the soil S fractionation method with analyses of stable isotopes of S and in situ soil solution studies will complete our knowledge on S biogeochemistry.

## Abbreviations

*RAS*: Readily available sulfate-S
*AS*: Adsorbed sulfate-S
*HCS*: HCl-soluble sulfate-S
*OS*: Organic sulfur
*ToS*: Total sulfur
*ES*: Ester sulfate-S
*CS*: Carbon bonded sulfur
*HIS*: HI-reducible sulfur
*PES*: Phosphate-extractable sulfate-S
*HCE*: HCl-extractable sulfate-S
